# Newly Synthesized Imino-Derivatives Analogues of Resveratrol Exert Inhibitory Effects in Breast Tumor Cells

**DOI:** 10.3390/ijms21207797

**Published:** 2020-10-21

**Authors:** Domenico Iacopetta, Rosamaria Lappano, Annaluisa Mariconda, Jessica Ceramella, Maria Stefania Sinicropi, Carmela Saturnino, Marianna Talia, Francesca Cirillo, Fabio Martinelli, Francesco Puoci, Camillo Rosano, Pasquale Longo, Marcello Maggiolini

**Affiliations:** 1 Department of Pharmacy, Health and Nutritional Sciences, University of Calabria, Via P. Bucci, 87036 Arcavacata di Rende, Italy; domenico.iacopetta@unical.it (D.I.); rosamaria.lappano@unical.it (R.L.); jessicaceramella@gmail.com (J.C.); marianna.talia@unical.it (M.T.); francesca.cirillo@unical.it (F.C.); francesco.puoci@unical.it (F.P.); marcello.maggiolini@unical.it (M.M.); 2 Department of Science, University of Basilicata, Viale dell’Ateneo Lucano 10, 85100 Potenza, Italy; annaluisa.mariconda@unibas.it (A.M.); fabiomartinelli@alice.it (F.M.); 3 Department of Biology and Chemistry, University of Salerno, Via Giovanni Paolo II, 132, 84084 Fisciano, Italy; plongo@unisa.it; 4 Biopolymers and Proteomics IRCCS, Ospedale Policlinico San Martino–IST, Largo R. Benzi 10, 16132 Genova, Italy; camillo.rosano@gmail.com

**Keywords:** resveratrol, resveratrol analogues, breast cancer, antitumor activity, topoisomerases, apoptosis, bioavailability

## Abstract

Breast cancer represents the most frequently diagnosed malignancy in women worldwide. Various therapeutics are currently used in order to halt the progression of breast tumor, even though certain side effects may limit the beneficial effects. In recent years, many efforts have been addressed to the usefulness of natural compounds as anticancer agents due to their low toxicity. Resveratrol, a stilbene found in grapes, berries, peanuts and soybeans, has raised a notable interest for its antioxidant, anti-inflammatory, and antitumor properties. Here, we report the design, the synthesis and the characterization of the anticancer activity of a small series of imino *N*-aryl-substituted compounds that are analogues of resveratrol. In particular, the most active compound, named **3**, exhibited anti-tumor activity in diverse types of breast cancer cells through the inhibition of the human topoisomerase II and the induction of apoptotic cell death. Therefore, the abovementioned compound maybe considered as a promising agent in more comprehensive treatments of breast cancer.

## 1. Introduction

**Figure 1:**
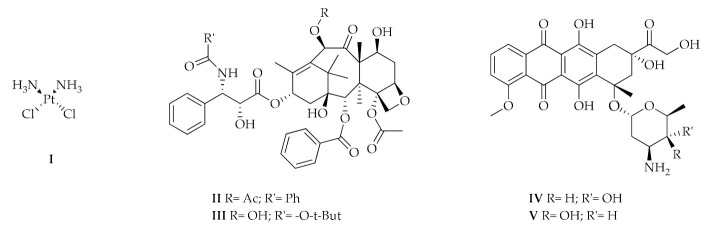
Molecular structures of Cisplatin (**I**), Paclitaxel (**II**), Docetaxel (**III**), Doxorubicin (**IV**), and Epirubicin (**V**).

Breast cancer represents the most diagnosed malignancy among women worldwide and the second cause of tumor death [1]. Therefore, numerous molecules have been designed, synthesized, and studied for their promising activity as anti-cancer agents. The pharmacological treatments are mainly based on the following compounds used alone or in combination (Figure 1):

(1) Nitrogen mustards, such as cyclophosphamide whose action depends on the alkylating ability of the active metabolite on the nitrogenous bases of DNA, in particular guanine;
(2) Platinum coordination complexes, such as Cisplatin (**I**), which can bind the nitrogenous bases of DNA forming cross-link bonds;
(3) Taxanes as Paclitaxel (**II**) and Docetaxel (**III**);
(4) Anthracycline like Doxorubicin (**IV**) and Epirubicin (**V**);
(5) Folic acid analogues as Methotrexate, which reversibly and competitively inhibits the dihydrofolate reductase (DHFR); and
(6) Pyrimidine analogues, in particular 5–fluorouracile [2].

**Figure 2:**
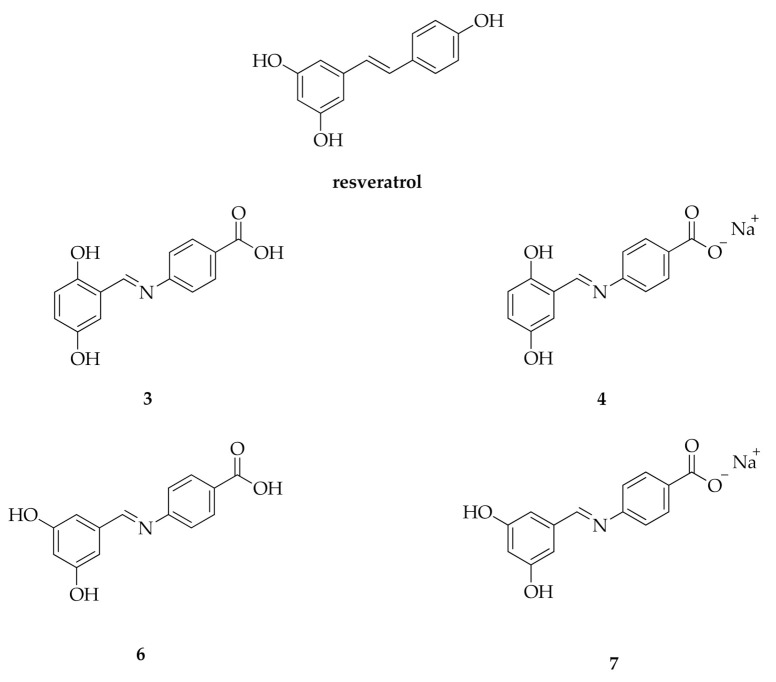
Structure of resveratrol and compounds **3**, **4**, **6**, and **7**.

A large body of studies has been performed in order to characterize the mechanisms of action of the above-mentioned compounds. Certain agents are able to inhibit the human topoisomerases I and II (hTopoI and II), crucial enzymes involved in the DNA replication, transcription, recombination and chromatin remodeling [3,4,5,6]. For instance, cisplatin is known for its ability to interact with many types of proteins regulating the DNA replication and cell division like DNA topoisomerase II [7]. However, cisplatin and other platinum complexes elicit considerable side effects [8,9,10,11,12]. The topoisomerases poisons anthracyclines, named doxorubicin and epirubicin, are currently effective chemotherapeutics used for the breast cancer therapy. Unfortunately, these compounds exert cardio-toxicity in long-term treatment. It has also been demonstrated that doxorubicin can trigger drug resistance, hence resulting in a poor prognosis and survival of patients [13,14,15,16,17]. In the metastatic breast cancer, paclitaxel and docetaxel are used, due to their ability to block the microtubules assembly and the topoisomerases activity. The presence of several adverse effects including nausea, vomiting, hair loss, and allergic reactions, may limit their therapeutic usefulness [18]. On the basis of the aforementioned observations, the identification of new compounds halting breast cancer without relevant side effects is strongly required. In this regard, numerous studies have evaluated through diverse in vitro and in vivo models the action of bioactive phytochemicals as preventive and therapeutic agents in breast cancer [19,20,21,22]. For instance, a member of the stilbene family, the polyphenol resveratrol (3, 4′,5-trihydroxy-stilbene, Figure 2) has recently drawn considerable attention for its benefits on human health due to its antioxidant, anti-inflammatory and anticancer action. Resveratrol, which is largely present in grapes, berries, peanuts and soybeans, can rely on the *cis*- and *trans*-configuration or the glycosylated form [23]. The anticancer properties of resveratrol are mainly linked to the ability to inhibit the hTopoII and to interfere with signaling pathways triggering the tumor development [24,25,26,27]. Regardless these relevant biological properties, resveratrol shows low solubility, short half-life, rapid clearance, low bioavailability and rapid metabolism, which cumulatively limit its clinical usefulness [28,29]. 

Here, we report the synthesis and the characterization of the antitumor activity of four hydroxy-benzene derivatives and resveratrol analogues, bearing an imino group *N*-aryl-substituted (Figure 2) [30]. In particular, the most active compounds (**3** and **4**) displayed antiproliferative activity in two breast cancer cell lines (MCF-7 and SkBr-3), without cytotoxic effects in a non-tumoral cell line (HEK293). Moreover, in silico and in vitro studies assessed the capability of these agents to inhibit the hTopoII, as observed upon resveratrol exposure.

## 2. Results

### 2.1. Chemistry

**Scheme 1:**
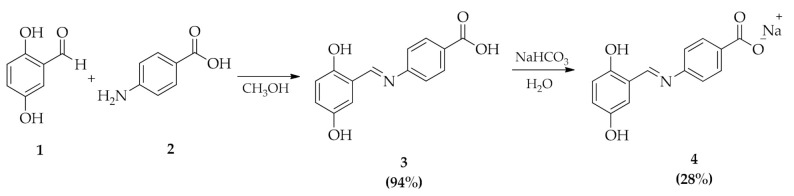
Synthesis of (E)-4-(2,5-dihydroxybenzylidene)-imino-benzoic acid (**3**) and (E)-4-(2,5-dihydroxybenzylidene)-imino-sodium benzoate (**4**).

**Scheme 2:**
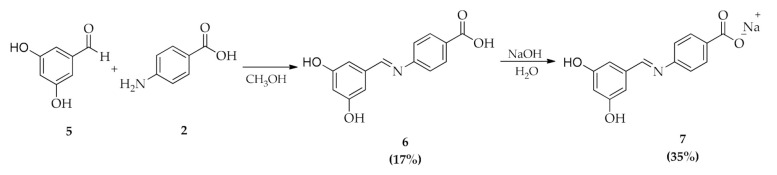
Synthesis of (E)-4-(3,5-dihydroxybenzylidene)-imino-benzoic acid (**6**) and (E)-4-(3,5-dihydroxybenzylidene)-imino-sodium benzoate (**7**).

The synthesis of (E)-4-(2,5-dihydroxybenzylidene)-imino-sodium benzoate (**4**) (Scheme 1) was achieved by reaction of 2,5-dihydroxybenzaldehyde (**1**) and 4-aminobenzoic acid (**2**) in methanol at reflux [31]. Then, the solvent was removed in vacuum and the (E)-4-(2,5-dihydroxybenzylidene)-imino-benzoic acid (**3**) was recovered as a dusty orange solid (yields 94%). Sodium salts (E)-4-(2,5-dihydroxybenzylidene)-imino-sodium benzoate (**4**) was obtained by reaction of (**3**) with sodium bicarbonate in aqueous solution with a yield of 28%.

(E)-4-(3,5-dihydroxybenzylidene)-imino-benzoic acid (**6**) was obtained by reaction of 3,5-dihydroxybenzaldeyde (**5**) with 4-aminobenzoic acid (**2**) in methanol (Scheme 2). The solution was stirred and refluxed for 24 h. After evaporation of the solvent in vacuum, a solid obtained was washed several times with CHCl_3_ to afford the pure product (**6**), yield 17%. (E)-4-(3,5-dihydroxybenzylidene)-imino-sodium benzoate (**7**) was obtained from compound **6** by reaction, for 20 min at room temperature, with a sodium hydroxide solution in deionized water (Scheme 2). The resulting suspension was filtered and the solid washed with CHCl_3_, yield 35%. All products were characterized by ^1^H and ^13^C NMR and mass spectroscopy (see experimental section). 

^1^H and ^13^C NMR chemical shifts are consistent with the proposed structures. In the NMR spectra of compounds (**3**), (**4**), (**6**), and (**7**), signals related to the imminic group are diagnostic. In the ^1^H NMR the signals of the imminic proton (CH = N) of the four compounds are attributed to 8.87, 8.83, 9.60, and 8.48 ppm, respectively. In the ^13^C NMR the peak of the imminic carbon (*C*H = N) is attributable to 164.2, 162.7, 162.6 and 160.5 ppm, respectively. The FT-IR spectra of (**3**) and (**4**) are measured in the 4000–400 cm^−1^ range. In both cases, in the FT-IR spectra are observed the absorbance of –OH groups, broad peak, at 3295 cm^−1^ for (**3**) and 3272 cm^−1^ for (**4**). The C = O stretching vibration appeared as a strong band with high intensity in the wavenumber region 1870–1540 cm^−1^. The corresponding peaks were observed at 1784 cm^−1^ for (**3**) and at 1736 cm^−1^ for (**4**). The C = N stretching (around 1690–1640 cm^−1^) are observed at 1675 cm^−1^ for (**3**) and at 1654 cm^−1^ for (**4**). In general, the aromatic C-H in-plane bending frequencies arise in the region 1500-1100 cm^−1^. The C-H in plane bending were observed at 1502, 1462, 1377, 1314, 1273, 1203, 1174, 1150, 1113 cm^−1^ for (**3**) and 1461, 1378, 1284, 1175, 1067 cm^−1^ for (**4**). Finally, the C-H bending of aromatic 1,2,4-trisubstituted rings appear at 790, 814 and 864 cm^−1^ for (**3**) and at 797, 784, 724 cm^−1^ for (**4**).

### 2.2. Bioavailability

In vitro bioavailability studies of compounds **3**, **4** (the salt of compound **3**) and resveratrol were performed in solutions simulating gastric and intestinal environment through a modified version of the traditional technique of dialysis membranes. The method of the dialysis membrane is divided in two enzymatic digestion phases: A first phase in which the action of pepsin occurs and a second phase in which participates the pancreatin (see experimental section). Bioavailability is defined as the percentage of analyte recovered in the bio-accessible fraction after in vitro digestion, in relation to the original undigested sample, as calculated according to the following equation [32]:

*Bioavailability (%)* = (Bioaccessible content/Total content) × 100

The results obtained are reported in Table 1.

These results highlight an improvement in the bioavailability in vitro of molecules **3** and **4** compared to resveratrol. After 2 h, the 16% and 17% of compounds **3** and **4,** respectively, were recovered in the bio-accessible fraction, while the 31% and 28% were recovered after the pancreatin digestion, hence reaching total value of 47% and 45% for **3** and **4**, respectively. As it concerns resveratrol, the total value was 35%.

### 2.3. Inhibitory Activity

In order to first investigate the effects of compound (**4**) on the proliferation of breast cancer cells, we used the MCF-7 and SkBr3 cells as model system. Performing MTT assay, we determined that this chemical exerts a cytotoxic activity with IC_50_ values of 50 µM ± 4 SD and 62 µM ± 6 SD in MCF-7 and SkBr3 cells, respectively. On the contrary, compounds **4** did not elicit anti-proliferative effects in non-tumoral HEK293 cells (data not shown). Then, we evaluated the DNA fragmentation by TUNEL assay. MCF-7 and SkBr3 cells exposed to 50 µM of compound **4** for 48 h were positive for TUNEL staining (data not shown), suggesting that this chemical exerts pro-apoptotic responses in breast tumor cells. On the basis of these findings, we aimed to extend the investigation to analogues of compound **4**, namely compounds **3**, **6**, and **7**. Hence, cells were treated for 48 h with these chemicals and resveratrol, which is known to exert anti-cancer properties in different breast cancer models [33]. Only compound **3** exerted an antiproliferative activity in the breast cancer cells used, while it did not elicit inhibitory effects in HEK293 cells (Table 2 and Supplementary Figure S1).

**Figure 3:**
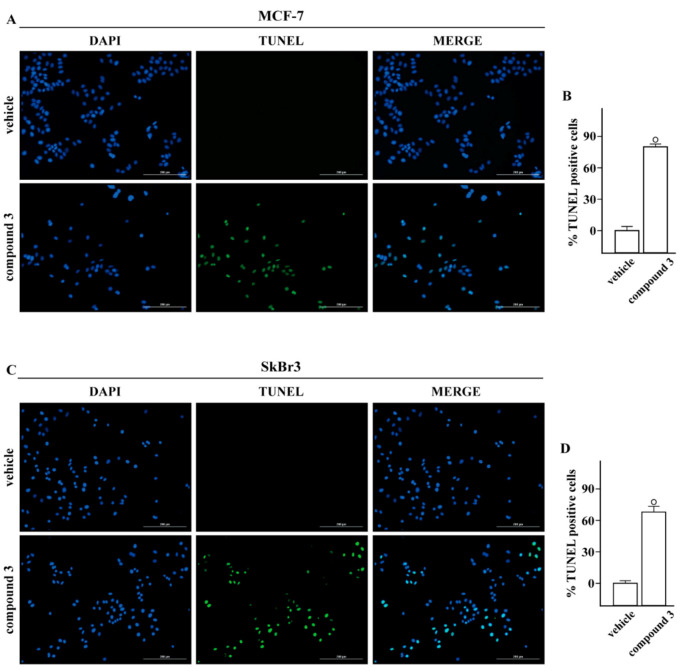
TUNEL assay. Compound **3** induces apoptotic cell death. TUNEL staining (green) in MCF-7 (**A**) and SkBr3 (**C**) cells treated for 24 h with vehicle or 10 μM **3** (compound 3), as indicated. Nuclei were stained by DAPI (blue). Each experiment shown is representative of 20 random fields observed. (**B**,**D**) Percentages of TUNEL-positive cells over vehicle-treated cells, (◦) indicates *p* < 0.05 for cells receiving vehicle versus treatments. Scale bar 200 μm.

In order to provide further insights into the biological action of compound **3**, we evaluated its potential to elicit pro-apoptotic effects in breast tumor cells. The treatment for 24 h with 10 μM of this compound increased the percentage of MCF-7 (Figure 3A,B) and SkBr3 (Figure 3C,D) TUNEL-positive cells, suggesting that compound **3** may display anti-proliferative and pro-apoptotic effects in breast cancer cells used.

### 2.4. Docking Studies

It is known that the antiproliferative properties of many drugs often depend on the inhibition of key enzyme(s) as for instance topoisomerases, telomerases, integrases, heparanase, and protein-kinases. In this context, DNA hTopoI and II represent valuable targets in order to interfere with the enzyme-DNA complexes toward DNA damages and consequently cell death.

**Figure 4:**
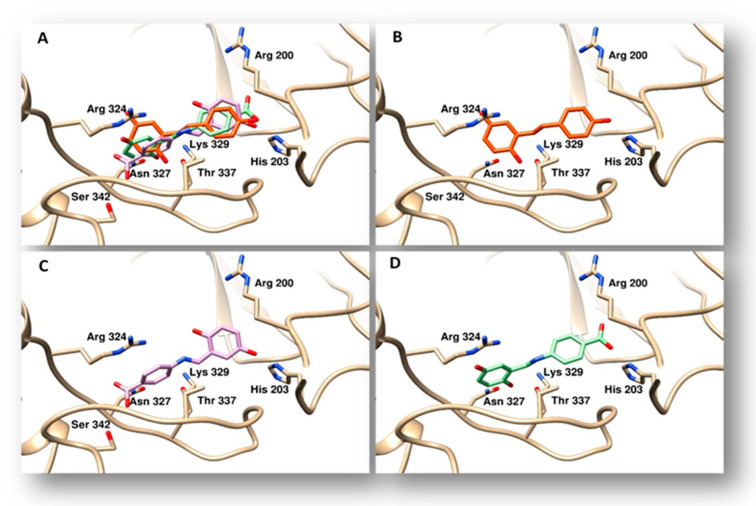
Binding modes of compounds evaluated to topoisomerase I. The protein is represented as tan ribbons, amino acids involved in the binding are evidenced as sticks and properly labelled. Panel (**A**), superposition of resveratrol, compounds **3** and **4**. Panel (**B**), the binding mode of resveratrol is indicated by orange sticks. Panel (**C**), binding mode of compound **3** drawn in violet sticks. Panel (**D**), compound **4** is reported as light green sticks in its binding pose.

**Figure 5:**
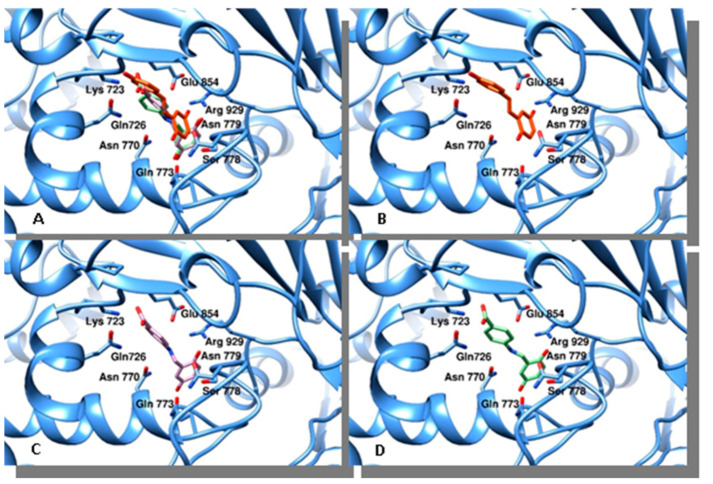
Binding modes of compounds evaluated to topoisomerase II. The protein is represented as cyan ribbons, amino acids involved in the binding are evidenced as sticks and properly labelled. Panel (**A**), superposition of resveratrol, compounds **3** and **4**. Panel (**B**), resveratrol is drawn in orange. Panel (**C**), compound **3** is shown as violet sticks. Panel (**D**), compound **4** is drawn as light green stick.

Aiming to assess whether resveratrol, compounds **3** and **4** could act as topoisomerases inhibitors, we performed docking studies on topoisomerases I and II (Figure 4 and Figure 5). Using as a target the three-dimensional coordinates of the hTopoI (PDB code 1T8I) [34], we performed docking simulations by a blind docking approach (no “a priori” information about the protein binding site was provided to the system). The program identified a binding site within the DNA binding cleft for resveratrol and both chemicals **3** and **4**. Such a binding mode could interfere with the correct processing of the DNA, however the experimental results did not confirm these findings (data not shown). Thereafter, to evaluate the binding poses of compounds synthesized with hTopoII, we performed further docking simulations. Using the blind-docking approach, we discovered that these agents bind to the same site nearness to the DNA binding site, therefore they may interfere with the protein function. All compounds were found positioned in an identical orientation within the binding cleft, Table 3 shows the amino acids involved in the interactions with the different agents.

### 2.5. In Vitro hTopoI and II Inhibition Assays

**Figure 6:**
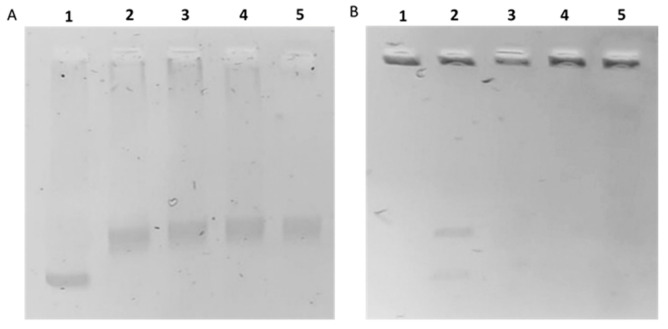
hTopoI and II assays. Panel (**A**): hTopoI relaxation assay. Supercoiled DNA (SCDNA) was incubated with or without hTopoI in the absence or presence of the tested compounds. Lane 1, SCDNA; lane 2, vehicle (DMSO); lane 3, 50 µM resveratrol; lane 4, 50 µM compound **3**; lane 5, 50 µM compound **4**. Panel (**B**): hTopoII decatenation assay. Kinetoplast DNA (kDNA) was incubated with hTopoII, with or without the tested compounds. Lane 1, kDNA; lane 2, vehicle (DMSO); lane 3, 50 µM resveratrol; lane 4, 50 µM compound **3**, lane 5, 50 µM compound **4**.

In order to confirm the in silico studies, we carried out the in vitro human topoisomerase I and II inhibition assays. DNA topoisomerases are important enzymes involved in the regulation of DNA replication, transcription, recombination, and in chromatin remodeling. These enzymes are vital for maintaining genomic integrity, thus DNA topoisomerases I and II represent good targets of anticancer drugs that, interfering with the enzyme-DNA complexes, produce permanent DNA damages leading to cell death [35,36,37,38,39]. As shown in Figure 6A, upon exposure to resveratrol, compounds **3** or **4**, no inhibition of hTopoI was observed (at least at the dose used in this assay, 50 μM), indeed the enzyme was able to form the relaxed DNA products (Figure 6A, lanes 3, 4, and 5). On the contrary, compounds **3** and **4** were able to inhibit the hTopoIIactivity (Figure 6B, lanes 4 and 5) and the intact kDNA migration on agarose gel failed very likely due toits large size. Similarly, resveratrol inhibited the hTopoII (Figure 6B, lane 3), as previously reported [25,26,40]. In the control experiment (Figure 6B, lane 2), the enzyme cut the kDNA and released the intact monomeric rings, which were notable at the bottom of the gel as two DNA bands representing the nicked open circular minicircles and the fully closed circular rings (decatenation products). Summing up, the compounds **3** and **4** elicited a selective inhibition of hTopoII.

## 3. Discussion and Conclusions

Breast cancer is the most common cancer in women and represents the second leading cause of cancer death in women worldwide [1]. To date, valuable meta-analyses have provided important knowledge on the usefulness of adjuvant chemotherapy to halt breast cancer recurrence and mortality [41]. In high-risk patients, both anthracyclines and taxanesare generally recommended. For instance, administration of doxorubicin and cyclophosphamide followed by paclitaxel treatment is a common regimen [42]. Platinum coordination complexes and pyrimidine analogues are also included in the pharmacological treatments of breast cancer. However, the usage of these well-known and effective drugs do exhibit severe side effects [43]. Along with toxic effects, long-term treatments may be involved in the onset of the multidrug resistance, leading to the failure of clinical responses [44]. In order to face these limitations, in recent years many researchers have focused their efforts toward alternative therapeuticsin breast cancer [45,46,47,48,49,50]. For instance, natural plant-derived bioactive compounds have obtained an increasing attention due to their anti-cancer abilities [51]. Natural compounds from dietary sources are known to interfere with several pathways involved in breast cancer initiation and progression [52,53]. It is worth mentioning that a large amount of anti-tumor drugs have been designed and developed from plant-derived ingredients [54]. In this context, previous data have reported that natural compounds may act in tumor cells modulating cell death signaling as the extrinsic and intrinsic apoptotic pathways, therefore suppressing the development of malignancies [55]. Resveratrol, which is a polyphenolic compound contained in dietary products like grapes, peanuts, soybeans, pomegranates and berries, has been recognized as a potent antioxidant, anti-inflammatory and chemopreventive agent able to interfere with different molecular targets [56]. On the basis of these observations, resveratrol could be considered as an effective agent in the treatment of various tumors as colorectal, liver, pancreatic, prostate, and breast cancers [57,58,59].The antitumor activities of resveratrol have been related to a plethora of signaling pathways that lead to cell cycle arrest, suppression of cell growth, apoptosis, reduction of inflammation and angiogenesis, inhibition of adhesion, invasion, and metastasis [60,61,62]. In this context, resveratrol has been shown to interfere with the growth of breast cancer cells prompting a cell-specific regulation of G1/S and G2/M phases of the cell cycle [63]. Among the diverse potential targets of resveratrol, it has been included a crucial enzyme for DNA replication and transcription named hTopoII [25,64]. Besides the acknowledged evidence supporting the beneficial anticancer effects of resveratrol, certain limitations may be considered as low bioavailability, short half-life, rapid clearance, low solubility and rapid metabolism [28].

Recently, many natural and synthetic analogs of *cis*- and *trans*-resveratrol have been designed and evaluated in order to improve the bioavailability and the solubility and to prevent the rapid metabolism [65,66]. In this regard, certain resveratrol derivatives were synthesized by substitutions of methoxy, hydroxyl, and other functional groups or through modifications of the double bonds [67]. Of note, these derivatives showed a stronger inhibitory activity in breast cancer cells respect to resveratrol as well as an improved bioavailability [67]. In addition, methoxylated analogues exhibited higher repressive effects in MCF-7 breast cancer cells than resveratrol [68]. 

The present investigation has added further data to the above-mentioned results through the synthesis and characterization of certain hydroxy-benzene derivatives bearing an imino group N-aryl-substituted (**3**, **4**, **6** and **7**). The antiproliferative activity of these new chemicals was evaluated in the estrogen receptor (ER)-positive MCF-7 and the estrogen receptor (ER)-negative SkBr3 breast cancer cells, as well as in the non-tumoral HEK-293 cells. Between the salt (E)-4-(2,5-dihydroxybenzylidene)-imino-sodium benzoate (**4**) and its precursor, the acid **3**, the last exhibited the highest antiproliferative activity with an IC_50_ values of 12 (± 1 SD) and 15 (± 1 SD) respectively in MCF-7 and SkBr3 cells. Worthy, both chemicals did not induce inhibitory effects in HEK-293 cells. Moreover, TUNEL assay showed a green nuclear fluorescence in MCF-7 and SkBr3 cells treated with compounds **3** and **4**, indicating the formation of damaged DNA and the consequent apoptotic response. 

Apoptosis is the process of programmed cell death that can be triggered by a wide variety of stimuli and conditions [69]. Chemicals able to inhibit the activity of some important enzymes, like topoisomerases, are effective inducers of apoptosis [70]. Aiming to assess whether compounds **3** and **4**, as well as resveratrol, could act as topoisomerases inhibitors, we performed docking studies on the hTopoI and II. In silico studies demonstrated that these compounds bind to both topoisomerases I and II closeness to the DNA binding site. However, experimental results indicated that compounds **3** and **4**, and resveratrol, inhibit the hTopoII, but not the hTopoI. Wealso determined the in vitro bioavailability of the compounds **3** and **4** and resveratrol, simulating gastric and intestinal environment through the use respectively of pepsin and pancreatin. Worthy, the molecules **3** and **4** exhibited a better bioavailability (47% and 45%, respectively) respect to resveratrol (35%).

In accordance with our previous studies [71], it could deserve interesting insights the evaluation of the structure-activity relationships of resveratrol and derivatives in cancer, in particular in breast tumor cells. In addition, the ability of certain chemicals to inhibit both the topoisomerase II and a main player of breast cancer progression like ER [72] may pave the way for next investigations toward innovative therapeutic approaches in breast tumor.

## 4. Materials and Methods

### 4.1. Chemistry

All reagents were purchased from Sigma–Aldrich (Milan, Italy). NMR deuterated solvents (Euriso-Top products) were kept in the dark over molecular sieves. NMR spectra were recorded with a Bruker AVANCE 400 operating at 400 MHz for ^1^H and at 100 MHz for ^13^C, respectively. The ^1^H NMR and ^13^C NMR chemical shifts are referred to SiMe_4_ (d = 0 ppm) using the residual proton impurities of the deuterated solvents as internal standards. Mass spectra (ESI) were obtained with a Waters Quattro Micro triple quadrupole mass spectrometer equipped with an electrospray ion source. Fourier transform infrared (FT-IR) spectra were obtained at a resolution of 2.0 cm^−1^ with a Bruker-Vector 22 FT-IR spectrometer equipped with a deuterated triglycine sulfate (DTGS) detector and Ge/KBr beam splitter. The frequency scale was internally calibrated to 0.01 cm^−1^ using a HeeNe reference laser. Thirty-two scans were signal-averaged to reduce spectral noise. 

The synthesis of (E)-4-(2,5-dihydroxybenzylidene)-imino-benzoic acid (**3**) was performed according to slightly modified literature procedure [31]. 1.0 g of 4-aminobenzoic acid (7.30 mmol) and 1.0 g of 4-hydroxybenzaldehyde (7.24 mmol) were dissolved in 60 mL methanol. The solution was stirred and refluxed for 12 h. After the reaction time an orange solution is obtained. The solvent removed under reduced pressure to afford the orange powder in good yield: **3** (1.75 g, 94%).

^1^H NMR (400 MHz, DMSO-d_6_): δ 11.88 (br, 1H), 8.87 (s, 1H), 8.00 (d, 2H), 7.44 (d, 2H), 7.08 (s, 1H), 6.89 (d, 1H), 6.81 (d, 1H). ^13^C NMR (100 MHz, DMSO-d_6_): δ 166.9, 164.2, 153.1, 152.5, 149.7, 130.6, 128.6, 121.7, 121.4, 119.3, 117.3, 116.6. ESI-MS (CH_3_CN, m/z): 258.5 Dalton [C_14_H_12_NO_4_]^+^, [MH]^+^. IR (KBr, nujol, cm^−1^): 3295_ν(–OH)_; 1784_ν(C=O)_; 1675_ν(CH=N)_; 1502, 1462, 1377, 1314, 1273, 1203, 1174, 1150, 1113, 790, 814, 864_ν(C-H)_.

Synthesis of (E)-4-(2,5-dihydroxybenzylidene)-imino-sodium benzoate (**4**): 300 mg of (E)-4-(2,5-dihydroxybenzylidene)-imino-benzoic acid (**3**) (1.07 mmol) were suspended in 38 mL of deionized water and stirred at room temperature for 15 min. Then, 42.3 mL of a 0.05 M solution of NaHCO_3_ were slowly dripped. The mixture was stirred for 8 h at room temperature. The brown suspension was filtered and the solvent removed in vacuo. The crude product was washed with methanol and dried in vacuo. The (E)-4-(2,5-dihydroxybenzylidene)-imino-sodium benzoate (4) was recovered as a brown dusty solid. (0.084 g, 28%).

^1^H NMR (400 MHz, DMSO-d_6_): δ 8.83 (s, 1H), 7.25 (d, 2H), 7.04 (s, 2H), 6.85 (d, 1H), 6.77 (d, 1H), 6.41 (d, 1H). ^13^C NMR (100 MHz, DMSO-d_6_): δ 169.3, 162.7, 153.0, 149.9, 148.7, 138.8, 130.2, 121.1, 120.1, 119.3, 117.1, 116.8. ESI-MS (CH_3_CN, m/z): 319 [C_14_H_11_NO_4_NaK]^+^. IR (KBr, nujol, cm^−1^): 3272_ν(–OH)_; 1736_ν(C=O)_; 1654_ν(CH=N);_ 1461, 1378, 1284, 1175, 1067, 797, 784, 724_ν(C-H)_.

Synthesis of (E)-4-(3,5-dihydroxybenzylidene)-imino-benzoic acid (**6**): 500 mg of 3,5-dihydroxybenzaldeyde (7.24 mmol) and 500 mg of 4-aminobenzoic acid (7.30 mmol) were solubilized in 35 mL of methanol and stirred at reflux for 24 h. Then, the solvent was removed in vacuo. The solid was washed in a Kumagawa extractor using refluxing chloroform for 5 h. The insoluble product was dissolved in methanol and then was filtered. The solvent was removed and the product (E)-4-(3,5-dihydroxybenzylidene)-imino-benzoic acid (**6**) is recovered as light brown-dusty solid (0.17 g, yield 17%).

^1^H NMR (400 MHz, DMSO-d_6_): δ 9.60 (s, 1H), 8.40 (s, 1H), 7.94 (d, 2H), 7.25 (d, 2H), 7.80 (d, 2H), 6.37 (d, 1H). ^13^C NMR (100 MHz, DMSO-d_6_): δ 167.5, 162.6, 158.7, 155.5, 137.5, 131.2, 130.6, 127.8, 121.0, 112.5, 108.6, 107.0, 106.2. ESI-MS (CH_3_CN, *m/z*): 258.6 [C_14_H_12_NO_4_]^+^.

Synthesis of (E)-4-(3,5-dihydroxybenzylidene)-imino-sodium benzoate (**7**): into a 50 mL one-neck flask was charged with 50 mg of compound **6** and 12.5 mL of deionized water. The suspension was stirred for 15 min at room temperature and 2.86 mL of a 0.05 M solution of NaOH were slowly dropped. The reaction was left under stirring for 5 min, filtered and the solvent removed in vacuo. The product was washed with chloroform (3.0 mL). The (E)-4-(3,5-dihydroxybenzylidene)imino-benzoate sodium (**7**) product was recovered as dark red vitreous solid (0.019 g, yield 35%).

^1^H NMR (400 MHz, DMSO-*d*_6_): δ 8.48 (s, 1H), 7.94 (d, 2H), 7.25 (d, 2H), 7.80 (d, 2H), 6.37 (d, 1H). ^13^C NMR (100 MHz, DMSO-*d*_6_): δ169.6, 160.5, 159.2, 158.7, 152.0, 138.1, 137.8, 130.8, 130.0, 19.7, 112.3, 108.7, 107.1, 106.6, 105.8. ESI-MS (CH_3_CN, *m/z*): 256.4 [C_14_H_10_NO_4_]^−^.

### 4.2. In Vitro Bioavailability Studies

In vitro bioavailability studies were performed according to the dialysis tubing procedure as previously reported in literature [32].

For this purpose, 100 µL of each tested compound (10 mM in DMSO) were employed and the concentration of each sample was determined using a UV/VIS spectrometer V-530 (Jasco, Cremella (LC), Italy).

The percentages were calculated according to the equations obtained from the prepared calibration curves at pH 1.0 and 7.0, respectively, for the two tested molecules.

The experiments were carried out in triplicate.

### 4.3. Docking Studies

The program Autodock v.4.2.2. [73] and the ADT graphical interface [74] were used to run the docking simulations that have been performed. We adopted a consolidated protocol we already used is several other cases for our docking simulations and that has been described in different of our previous publications [75,76]. As a target for all our simulations, we used the molecular structure of the human topoisomerase IIalpha in complex with DNA and etoposide [77] [PDB code 5GWK]. Figure 4 and Figure 5 were drawn with the program Chimera [78].

### 4.4. Biological Assays

#### 4.4.1. Cell Cultures

MCF-7, SkBr3 breast cancer cells and human embryonic kidney 293 (HEK293) cells were obtained by ATCC (Manassas, VA, USA), used less than 6 months after resuscitation and routinely tested and authenticated according to the ATCC suggestions. SkBr3 cells were maintained in RPMI-1640 (Life Technologies, Milan, Italy) without phenol red, supplemented with 5% fetal bovine serum (FBS) and 100 μg/mL penicillin/streptomycin (Life Technologies, Milan, Italy). MCF-7 and HEK-293 cells were maintained in DMEM (Dulbecco’s modified Eagle’s medium) (Life Technologies, Milan, Italy) with phenol red, with a supplement of 5% FBS and 100 μg/mL of penicillin/streptomycin.

#### 4.4.2. Cell Viability Assay

The effects of each compound on cell viability were determined by the MTT [3-(4,5-dimethylthiazol-2-yl)-2,5-diphenyltetrazolium bromide] assay, as previously reported [79]. Cells were seeded in quadruplicate in 96-well plates in regular growth medium and grown until 70–80% confluence. Cells were washed once they had attached and then treated with increasing concentrations of each compound for 1 day in regular medium supplemented with 2% FBS. Relative cell viability was determined by MTT assay according to the manufacturer’s protocol (Sigma-Aldrich, Milan, Italy). Mean absorbance for each drug dose was expressed as a percentage of the control untreated well absorbance and plotted versus drug concentration. IC_50_ values represent the drugs concentration able to reduce the cells viability of 50% respect to the untreated control cells (vehicle).

#### 4.4.3. TUNEL Assay

Cell apoptosis was determined by TdT-mediated dUTP Nick-End Labeling (TUNEL) assay, conducted using DeadEnd Fluorometric TUNEL System (Promega, Milan, Italy) and performed according to the manufacturer’s instructions. Briefly, cells were treated for 48 h, then were fixed in freshly prepared 4% paraformaldehyde solution in PBS (pH 7.4) for 25 min at 4 °C. After fixation, cells were permeabilized in 0.2% Triton X-100 solution in PBS for 5 min. After washing twice with washing buffer for 5 min, the cells were covered with equilibration buffer at room temperature for 5–10 min. The labeling reaction was performed using terminal deoxynucleotidyl transferase end-labeling TdT and fluorescein-dUTP cocktail for each sample and incubated for 1 h at 37 ◦C where TdT catalyzes the binding of fluorescein-dUTP to free 3′OH ends in the nicked DNA. After rinsing, cells were washed with 2 × SSC solution buffer and subsequently incubated with 4′,6-Diamidino-2-Phenylindole (DAPI, Sigma-Aldrich, Milan, Italy) to stain nuclei and analyzed using the Cytation 3 Cell Imaging Multimode Reader (BioTek, Winooski, VT, USA). Statistical analysis was done using ANOVA followed by Newman–Keuls’ testing to determine differences in means. *p* < 0.05 was considered as statistically significant.

#### 4.4.4. Human Topoisomerase I (hTopoI) Relaxation Assay

Human topoisomerase I relaxation assays were performed incubating the supercoiled pHOT1 (used as substrate) with the recombinant human topo I (TopoGEN, Port Orange, FL, USA) and all the tested compounds, according to the guidelines of the manufacturer (TopoGEN, Port Orange, FL, USA), with some modifications [37].

#### 4.4.5. Human Topoisomerase II (hTopoII) Decatenation Assay

Human topoisomerase II decatenation assays were carried out incubating the human topoisomerase II (TopoGEN, Port Orange, FL, USA) with kinetoplast DNA (used as substrate) and all the tested compounds, according to the guidelines of the manufacturer (TopoGEN, Port Orange, FL, USA), with some modifications [37].

## Figures and Tables

**Table 1:** In vitro bioavailability (%) of resveratrol, **3** and **4** after pepsin (2 h) and pancreatin (4 h) digestions.

	2 h pH 1.0	4 h pH 7.0	Total 6 h
**resveratrol**	12 ± 0.6	23 ± 0.8	35 ± 1.1
**3**	16 ± 0.7	31 ± 1.1	47 ± 1.6
**4**	17 ± 0.8	28 ± 0.9	45 ± 1.3

**Table 2:** Antiproliferative activity of tested compounds on breast MCF-7 and SkBr3, cancer cells and HEK293 cells, after 48 h treatment, as determined by using the MTT assay. IC_50_ values were calculated by probit analysis (*p* < 0.05, χ2 test) and are the mean ± SD of three independent experiments performed in triplicate.

IC_50_ (µM) ± S.D.			
Compound	MCF-7	SkBr3	HEK293
**3**	12 (±1)	15 (±1)	>90
**6**	>90	>90	>90
**7**	>90	>90	>90
**resveratrol**	>90	>90	>90

**Table 3:** Binding energies and residues involved in the interaction between topoisomerase II and compounds indicated.

Compound	Binding Energy (Kcal/mol)	Calculated Ki (μM) *	Topo II Alpha Residues Involved in Ligand Binding §	
**resveratrol**	**−6.73**	11.7	**Gln726, Asn770, Asn779, Glu854, Arg929**	***Pro716***
**3**	**−6.8**	10.4	**Lys723, Gln726, Asn770, Glu773, Arg929**	***Pro716***
**4**	**−7.25**	4.8	**Lys723, Gln726, Asn770, Glu773, Arg929**	***Pro716***

* Ki values as calculated by Autodock algorithm: Ki = exp(ΔG/(R*T). § Residues involved in polar interactions. Residues forming hydrogen bonds are listed in **bold**. Hydrophobic contacts in *Italic*.

## Supplementary Materials

The following are available online at https://www.mdpi.com/1422-0067/21/20/7797/s1, Figure S1: Evaluation of the antiproliferative response to compound **3**.

## Abbreviations

^13^C NMR	carbon nuclear magnetic resonance
^1^H NMR	proton nuclear magnetic resonance
ANOVA	analysis of variance
BSA	bovine serum albumin
d	doublet
DAPI	2-(4-amidinophenyl)-6-indolecarbamidine dihydrochloride
DHFR	dihydrofolate reductase
DMEM	Dulbecco’s modified Eagle’s medium
DMSO	dimethyl sulfoxide
DMSO-d6	deuterated dimethyl sulfoxide
DNA	deoxyribonucleic acid
dUTP	2′-deoxyuridine 5′-triphosphate
EB	ethidium bromide
EDTA	ethylenediaminetetraacetic acid
ER	estrogen receptor
ESI-MS	electrospray ionization mass spectrometry
FBS	fetal bovine serum
HEK-293	human embryonic kidney 293
hTopo	human topoisomerase
Hz	hertz
IC_50_	half maximal inhibitory concentration
IR	infrared
kDNA	kinetoplast DNA
m/z	mass/charge
MCF-7	michigan cancer foundation-7
MDA-MB-231	M.D. Anderson metastatic breast-231
MS (EI)	electron ionization mass spectrometry
MTT	3-(4,5-dimethylthiazol-2-yl)-2,5-diphenyltetrazolium bromide
PBS	phosphate-buffered saline
PDB	protein data bank
s	singlet
SCDNA	supercoiled DNA
SD	standard deviation
SkBr-3	Sloan–Kettering breast-3
TAE	tris-acetic acid-EDTA
TE	tris-EDTA
TUNEL	terminal deoxynucleotidyl transferase dUTP nick end labeling
U	units
UV	ultraviolet
Vis	visible
δ	chemical shift
