# Supplementary material for: Newly Synthesized Imino-Derivatives Analogues of Resveratrol Exert Inhibitory Effects in Breast Tumor Cells
Source: Int J Mol Sci. 2020 Oct 21;21(20):7797. doi: 10.3390/ijms21207797 (PMC7589783; doi:10.3390/ijms21207797)
Supplement: Supplementary file 1 [file ijms-21-07797-s001.pdf]

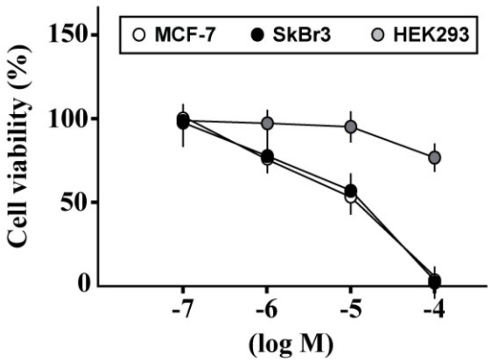

Supplementary Figure 1. Evaluation of the antiproliferative response to compound **3** in MCF-7 and SkBr3 breast cancer cells and in HEK293 cells upon a 48 h treatment, as determined by using the MTT assay. Cell viability was expressed as the percentage of cells treated with the different concentrations of compound **3** respect to cells treated with vehicle. Data shown are the mean ± SD of three independent experiments performed in triplicate.
